# Definition, etiology, prevention and treatment of peri-implantitis – a review

**DOI:** 10.1186/1746-160X-10-34

**Published:** 2014-09-03

**Authors:** Ralf Smeets, Anders Henningsen, Ole Jung, Max Heiland, Christian Hammächer, Jamal M Stein

**Affiliations:** 1 Department of Oral and Maxillofacial Surgery, University Medical Center Hamburg-Eppendorf, Martinistr. 52, 20246 Hamburg, Germany; 2 Private Practice, Schumacherstrasse 14, 52062 Aachen, Germany; 3 Department of Conservative Dentistry, Periodontology and Preventive Dentistry, University Hospital Aachen, Pauwelsstr.30, 52074 Aachen, Germany

**Keywords:** Peri-implantitis, Peri-implant disease, Review, Periodontal disease, Mucositis, Peri-implantitis therapy, Epidemiology, Etiology

## Abstract

Peri-implant inflammations represent serious diseases after dental implant treatment, which affect both the surrounding hard and soft tissue. Due to prevalence rates up to 56%, peri-implantitis can lead to the loss of the implant without multilateral prevention and therapy concepts. Specific continuous check-ups with evaluation and elimination of risk factors (e.g. smoking, systemic diseases and periodontitis) are effective precautions. In addition to aspects of osseointegration, type and structure of the implant surface are of importance. For the treatment of peri-implant disease various conservative and surgical approaches are available. Mucositis and moderate forms of peri-implantitis can obviously be treated effectively using conservative methods. These include the utilization of different manual ablations, laser-supported systems as well as photodynamic therapy, which may be extended by local or systemic antibiotics. It is possible to regain osseointegration. In cases with advanced peri-implantitis surgical therapies are more effective than conservative approaches. Depending on the configuration of the defects, resective surgery can be carried out for elimination of peri-implant lesions, whereas regenerative therapies may be applicable for defect filling. The cumulative interceptive supportive therapy (CIST) protocol serves as guidance for the treatment of the peri-implantitis. The aim of this review is to provide an overview about current data and to give advices regarding diagnosis, prevention and treatment of peri-implant disease for practitioners.

## Introduction

Dental implants have become an indispensable established therapy in dentistry in order to replace missing teeth in different clinical situations. Success rates of 82,9% after 16 years follow-up have been reported [1]. Under care and attention of indications, anatomical and intra-individual limiting factors, insertion of dental implants seems to represent a “safe” treatment option. Nevertheless, in the last decades increasing evidence raised on the presence of peri-implant inflammations representing one of the most frequent complications affecting both the surrounding soft and hard tissues which can lead to the loss of the implant. Therefore, strategies for prevention and treatment of peri-implant disease should be integrated in modern rehabilitation concepts in dentistry. The present review gives an updated overview on the pathogenesis, etiology, risk factors and prevention of peri-implantitis, but also on actual recommendations in treatment and therapy options.

## Review

### Definition und pathogenesis

In analogy to gingivitis and periodontitis affecting the periodontium of natural teeth, an inflammation and destruction of soft and hard tissues surrounding dental implants is termed as mucositis and peri-implantitis [2-4]. Thereby, transitions are often fluent and not clinically clearly separable [5].

**Figure 1:**
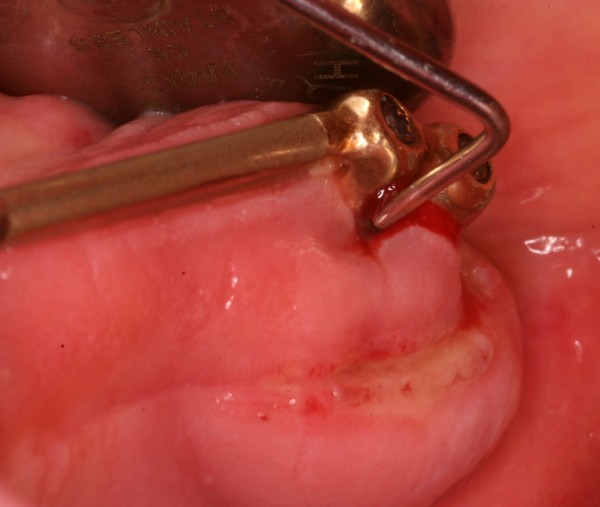
Peri-implantitis with increased probing depth (12 mm).

Mucositis describes a bacteria-induced, reversible inflammatory process of the peri-implant soft tissue with reddening, swelling and bleeding on periodontal probing (Figure 1) [2-6]. These are typical signs, but they are sometimes not clearly visible. Furthermore, bleeding on probing (BOP) might be an indicator for peri-implant disease, but sufficient evidence according to the predictive value of BOP is still lacking [7].

In contrast to mucositis, peri-implantitis is a progressive and irreversible disease of implant-surrounding hard and soft tissues and is accompanied with bone resorption, decreased osseointegration, increased pocket formation and purulence [2-6]. Bleeding on probing, bone loss and deep probing depths may have other reasons than inflammation, e.g. too deep insertion of the implant [8]. Moreover, type and shape oft the implant, connection type, abutment and suprastructure material and the type of prosthetic suprastructure affect the peri-implant soft and hard tissues [7].

Depending on the configuration of the bony defect, *Schwarz et al.* distinguished between an intraosseous class I defect and a supra-alveolar class II defect in the crestal implant insertion area [5]. *Spiekermann* characterized the type of bone resorption into horizontal (class I), key-shaped (class II), funnel- and gap-like (class III a, b) as well as horizontal-circular (class IV) forms [9]. However, it is not possible to conclude progression and prognosis criteria from these classifications.

On a microscopic and molecular level, striking differences between peri-implant tissue and intact periodontium can be determined (Table 1). Due to the reduced vascularization and parallel orientation of the collagen fibres, peri-implant tissues are more susceptible for inflammatory disease than periodontal tissues. This phenomenon can be verified immunohistochemically through increased formation of inflammatory infiltrate, nitric oxide 1/3, VEGF, lymphocytes, leukocytes and Ki-67 [10]. Besides, in analogy to periodontitis the level of matrix-metalloproteinases (MMP), such as MMP-8, is increased up to 971% in peri-implant lesions. The latter can be used for disgnostic purposes [11-13].

**Table 1:** **Comparison of peri-implant mucosa with physiological periodontium **[3,5]

**Peri-implant mucosa**	**Physiological periodontium**
Desmosomes and hemidesmosomes of epithelium and junctional epithelium (biological width) are linked with the contact surface	
Direct bone-to-implant contact	Anchoring system of root cementum, alveolar bone and desmodontic fibers
Subepithelially more collagen fibers and less fibroblasts/vessels	Subepithelially more fibroblasts and vessels
Parallel collagen fibers in relation to implant surface	Dentogingival, dentoperiostal, circular and transseptal fiber orientation

A differentiation of peri-implantitis to other inflammatory periodontal processes cannot be made on the basis of human saliva by markers such as osteocalcin, tartrate-resistant acid phosphatase (TRAP), dickkopf-related protein-1 (DKK-1), osteoprotegerin (OPG) and cathepsin K (CatK) [9,14].

### Etiology and epidemiology

There are several reports on the prevalence of mucositis and peri-implantitis that differ between 5% and 63.4%. This enormous range is mainly based on varying study designs and population sizes with different risk profiles and statistic profiles [5,15-18].

*Zitzmann et al.* quantified the incidence of the development of peri-implantitis in patients with a history of periodontitis almost six times higher than in patients with no history of periodontal inflammation [3]. After 10 years, 10% to 50% of the dental implants showed signs of peri-implantitis [19,20]. Based on the Consensus Report of the Sixth European Workshop in Periodontology, *Lindhe & Meyle* reported an incidence of mucositis of up to 80% and of peri-implantitis between 28% and 56% [21].

However, the prevalence of peri-implant diseases, evaluated recently by *Mombelli et al.*, revealed peri-implantitis in 20% of all implanted patients and in 10% of all inserted implants. Although this percentage has to be interpreted with caution because of the variability of the analyzed studies [7], it underlines the fact that bone remodeling processes often result in marginal bone loss during the first weeks after abutment connection which cannot be regarded as peri-implantitis. This led to the recommendation to take a radiograph after insertion of the suprastructure and to consider it as a basis for any future assessment of peri-implant bone loss.

Frequently, a spectrum of pathogenic germs can be detected such as *Prevotella intermedia, Prevotella nigrescens, Streptococcus constellatus, Aggregatibacter actinomycetemcomitans, Porphyromonas gingivalis, Treponema denticola* and *Tannerella forsythia*[3,22]. *Rams et al.* revealed 71.7% resistance to at least one antimicrobial substance in a group of 120 patients [22]. Peri-implantitis is a poly-microbial anaerobic infection [23]. However, in contrast to periodontitis, peri-implantitis lesions harbor bacteria that are not part of the typical periodontopathic microbiota. In particular, *Staphylococcus aureus* appears to play a predominant role for the development of a peri-implantitis. This bacterium shows an high affinity to titanium and has according to the results of *Salvi et al.* a high positive (80%) and negative (90%) predictive value [24]. As another beneficial cause, smooth implant surfaces in comparison to rough surfaces can accelerate the peri-implant inflammation [10,17,25].

### Risk factors and prevention

Implant loss may occur as “early implant loss” up to one year after implant insertion and “delayed implant loss” with a time period of more than one year after implant insertion [3]. The following factors or circumstances have been reported as risk factors for the development of peri-implantitis [5,6,16,26-33]:

• Smoking with additional significantly higher risk of complications in the presence of an positive combined IL-1 genotype polymorphism.

• History of periodontitis.

• Lack of compliance and limited oral hygiene (including missing checkups).

• Systemic diseases (e.g. maladjusted diabetes mellitus, cardiovascular disease, immunosuppression).

• Iatrogenic causes (e.g. “cementitis”).

• Soft tissue defects or poor-quality soft tissue at the area of implantation (e.g. lack of keratinized gingiva).

• History of one or more failures of implants.

Studies indicate smoking as the greatest identifiable and most often cited risk factor for peri-implant disease followed by a history of periodontitis. Both are related to higher prevalences of peri-implantitis [7]. The presence of periodontitis or cigarette smoking increased the risk for peri-implantitis up to 4.7-fold as reported by *Wallowy et al.*[6]. Moreover, smoking has been shown to be a predictor for implant failure [31]. In a recent meta-analysis smoking increased the annual rate of bone loss by 0.16 mm/year and represented the main systemic risk factor [34]. The extent of osseointegration as well as the oral hygiene around dental implants was found to be reduced among smokers [35]. It is commonly accepted that the outcome of almost all intraoral therapeutic parameters are negatively affected by smoking although not in all previous studies a positive correlation between peri-implantitis and tobacco smoking could be found [36,37]. Evidence of predictors for implant success such as gender or age could not be found but for the jaw of treatment (maxillary versus mandibular implants). In a study by *Vervaeke et al.* maxillary implants were at a significantly higher risk for peri-implant bone loss compared to mandibular implants [31]. Bone augmented areas could not be determined as risk factors for implant failure or increased peri-implant disease [38].

Across an observation period of 10 years in a group of patients with periodontitis, the previously eliminated bacterial strains of *Aggregatibacter actinomycetemcomitans* and *Porphyromonas gingivalis* could again be detected in the oral mucosa [3]. *Prevotella intermedia* was, however, continuously evident. This indicates a niche survival of bacteria after tooth extraction with recurrence of the same microflora after a short period of time. In particular, attention should be paid to the remaining teeth with periodontitis as a potential source of infection. Therefore, the type of dentition (edentulous versus partially edentulous) may influence the colonization of peri-implant tissues with periodontal pathogens [38].

The impact of keratinized gingiva around dental implants has been controversially discussed, but most studies emphasize the importance of an adequate zone of keratinized tissue surrounding implants [39-41]. The so called “cementitis” may be regarded as the most important identifiable iatrogenic risk factor since its first description by *Wilson et al.* in 2009 [42]. The latter group revealed that residual dental cement in a group of patients with clinical or radiographic signs of peri-implant disease was present in 81% of the sites. After its removal, clinical signs were absent in 74% of the affected sites. *Korsch et al.* found that the removal of cement remnants led to a decrease of the inflammatory response by almost 60% [43]. *Linkevizius et al.* examined the manifestation of peri-implantitis in a group of patients with present cement remnants. In those who had a history of periodontitis, peri-implantitis was found in 100% of the patients, whereas cement remnants in patients with no previous periodontal disease ended up in 65% peri-implantitis manifestations [30]. Another preventive arrangement regarding antibacterial precautions are internal connections with inward located microgap, which should be preferred. [6].

Peri-implant probing is recommended to be carried out carefully with a minimal probing force. However, the so-called *platform switch* (abutment is located horizontally between implant and crown) can complicate probing and, thus, hide the true extension of peri-implantitis [3,5,17,26,44]. Nevertheless, studies have indicated that platform switch might be an important protective factor against peri-implant disease [45].

Implant loss can be differentiated on the basis of the following additional factors [3,5,6,46-49]:

• Overloading of the implant,

• Faults in material and techniques,

• Poor bone quality at the implant area,

• Systemic diseases and drug therapies, which inhibit bone modulations according to “Wolff’s law” (bone density and strength increase with stress - and vice versa).

Thus, implants of more than 10 mm length in square thread design show higher success rates than shorter implant lengths or shapes without thread or buttress thread [48,49]. Also rough implant surfaces of more than 2 microns seem to feature better osseointegration than smooth (<0.5 microns) or moderate surfaces (1–2 microns) [17].

Development of strengths in the temporomandibular joint of more than 1300 Newton may shift the implants in the first few months of healing up to 100 microns by presence of sagittal forces acting from an average of 50 Newton [46]. These average reference forces even increase to 87 Newton when articulation angles up to 60° in horizontal axis.

In addition to patient training sessions for optimal oral hygiene, preventive strategies such as professional tooth and implant cleaning as well as individually continuous peri-implant examinations (probing status) should be considered in order to prevent peri-implant diseases (Table 2) [6]. Attention has to be paid, in particular, to the reduction of the above-mentioned risk factors such as heavy smoking or diabetes mellitus.

**Table 2:** Numbers of check-ups (cu) annually for different patient collectives

	**cu = 1**	**cu = 2**	**cu > 3**
Oral hygiene and hygienic ability of the implant	well	middle	bad
Smoking status	/	in history	in presence
Periodontitis, mucositis (with history)	/	/	in presence
Other risk factors	/	/	e.g. systemic diseases, history of an non-successful implant insertion

As part of a holistic therapy, so-called reference parameters (“hour zero”) and clearly determined control procedures have to be assessed with adequate documentation. Radiographs should be taken pre-, intra- and post operatively in order to get information about the implantation site in which peri-implant inflammation will be detectable as brightening zones indicating increased bone resorption [6].

Prevention of peri-implant disease starts with a sufficient and structured planning including individual evaluation and minimization of risk factors (smoking, compliance, oral hygiene, periodontal disease, systemic diseases), establishment of optimal soft and hard tissue conditions, the choice of the correct implant design followed by a maximally atraumatic approach and regular clinical examinations with a periodontal probing status.

### Therapy

The treatment of peri-implant infections comprises conservative (non-surgical) and surgical approaches. Depending on the severity of the peri-implant disease (mucositis, moderate or severe peri-implantitis) a non-surgical therapy alone might be sufficient or a step-wise approach with a non-surgical therapy followed by a surgical treatment may be necessary.

#### ***Therapy of mucositis***

One of the main aims of peri-implant therapy is to detoxify the contaminated implant surface. In the presence of peri-implant mucositis, non-surgical methods are appropriate and sufficient for detoxification. These include mechanical implant cleaning with titanium or plastic-curettes, ultrasonics or air polishing. Moreover, photodynamic therapy as well as local antiseptic medication (chlorhexidinglukonate, hydrogen peroxide, sodium percarbonate, povidone-iodine) may support the antimicrobial therapy.

In two randomized clinical trials *Heitz-Mayfield et al. and Hallström et al.* were not able to prove any benefits in reduction of pocket depth, plaque index or purulency when adjuvant antimicrobial therapy (chlorhexidine and azithromycine) was used in addition to mechanical therapy only [50,51]. Reductions of the bleeding index were explained by the general improvement of oral hygiene with reference to the potential importance of guidelines and treatment protocols [50-52]. The establishment of an adequate oral hygiene should, therefore, be considered as key issue of the prevention of peri-implant infections. Besides, a maintenance program with regular evaluation of the peri-implant probing depths, supportive professional implant cleaning and oral hygiene training should be integral part of every post-operative care after implant insertion [2,6].

#### ***Therapy of peri-implantitis***

Most of the published strategies for peri-implantitis therapy are mainly based on the treatments used for teeth with periodontitis. The reason is that the way of bacterial colonization of dental and implant surfaces follow similar principles, and it is commonly accepted that the microbial biofilm plays an analogous role in the development of peri-implant inflammation [53]. For the treatment of peri-implantitis, both conservative (non-surgival) as well as surgical therapies can be applied. Thereby, the surgical treatments can be done using resective or regenerative approaches [54-59].

#### ***Conservative therapy***

In addition to medication and manual treatment (e.g. with curettes, ultrasonic and air polishing systems) innovative techniques such as laser-supported and photodynamic therapy methods are recently described as conservative therapy options.

#### ***Manual treatment***

**Figure 2:**
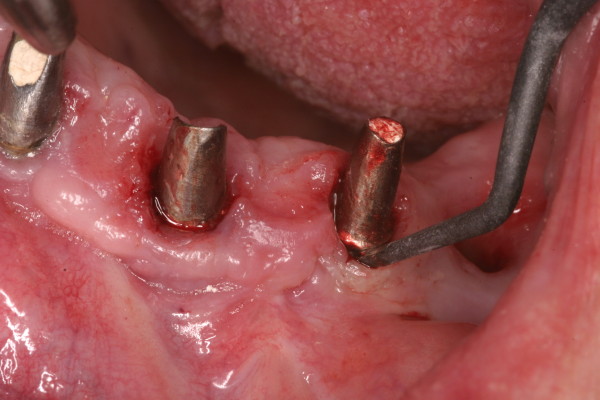
Conservative therapy – example of the use of a carbone curette.

Basic manual treatment can be provided by teflon-, carbon-, plastic- and titanium curettes (Figure 2).

Due to the fact that therapy with conventional curettes is able to modify the implant surface and can roughen the surface, it has been recommended that the material of the tip should be softer than titanium [60,61]. It is possible to reduce bleeding on probing scores by cleaning with piezoelectric scalers as well as with hand instruments, and no differences have been found between these methods concerning reduction of bleeding on probing, plaque index and probing depths after at least 6 months [62,63].

**Figure 3:**
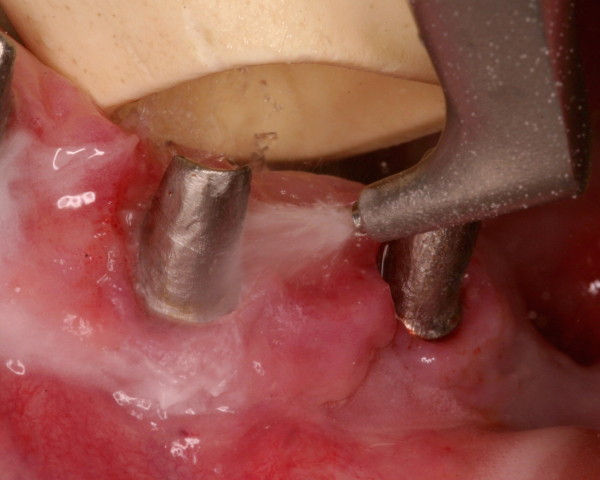
Conservative therapy – detoxification using an air polishing device with glycin powder.

As to the above-mentioned methods, the efficacy of ultrasonic curettage seems to underly the use of air polishing systems (Figure 3) [5,62,64-68]. *Persson* et al. and *Renvert et al.* experienced significantly lower numbers of bacteria with partial reduction of plaque and bleeding scores after mechanical curettage, while *Schwarz et al.* reported 30%-40% less residual biofilm areas by using ultrasonic methods [5,63,66]. Depending on the surface topography of the implants, *Louropoulou et al.* recommend different therapeutic methods (Table 3).

**Table 3:** **Qualitative effectiveness (x: yes/o: no) of different cleaning methods depending on implant surface **[68]

	**Smooth surface**	**Sandblasted and acid-etched surface (SLA)**	**Plasma sprayed surface**
**Rubber cap**	o	o	o
**Metalic curette, rotating titanium brush**	o	x	x
**Plastic curette**	o	o	o
**Ultrasonic systems with metalic tips**	x (polished)		
**Ultrasonic systems with plastic tips**	o	x	x
**Air polishing**	x	x	x

The results of air polishing systems are depending on the used medium and are significantly better in the following order: hydroxylapatite/tricalcium phosphate > hydroxylapatite > glycine > titanium dioxide > water and air (control group) > phosphoric acid [64].

An abrasive air polishing medium can modify the surface of implants. After air powder treatment cell attachment and cell viability still showed sufficient levels, but cell response was decreased compared with sterile surfaces [64,65,67]. The extent of re-osseointegration of titanium implants after air polishing therapy has been reported between 39% and 46% with increased clinical implant attachment and pocket depth reduction [65]. The occurrence of bleeding on probing, one of the qualitative parameters in the presence of a peri-implantitis, could be significantly reduced [67].

#### ***Drug therapy***

There are numerous in vitro and in vivo studies on the application of medicaments as part of the treatment of mucositis and peri-implantitis. However, due to differences in the design of all studies, comparison of these studies is difficult. The following therapies can be distinguished:

• Antiseptic rinses in relation to different parameters.

• Application of systemic and locally delivered antibiotics in relation to pocket depth or different parameters.

In a review by *Javed et al.,* summarizing nine studies, systemic and local antibiotic applications (e.g. tetracycline, doxycycline, amoxicillin, metronidazole, minoxicycline hydrochloride, ciprofloxacin, sulfonamides + trimethoprim) led to significant reductions of pocket depths in a period between one and six years [69]. *Moura et al.* noticed the same for resorbable doxicycline releasing nanospheres in local application over a period of 15 months [70]. *Leonhardt et al.* noticed an overall success rate of 58% when treating peri-implantitis with surgical debridement and the use of various antibiotics and combinations of them (including clindamycin, amoxicillin + metronidazole, tetracycline, ciprofloxacin) [71].

*Astasov-Frauenhoffer et al.* were able to prove complete growth-inhibiting effects of amoxicillin and metronidazole on *Streptococcus sanguinis*, *Porphyromonas gingivalis* and *Fusobacterum nucleatum* apart from each other, but the combination was found to be more efficient than metronidazole alone [72]. Comparing local antibiotic therapy with photodynamic therapy, *Bassetti et al.* presented no differences in reduction of pocket depths or reduction of the number of bacteria in the periodontal pockets [73]. Grapefruit juice, known as antioxidant, had only a bacteriostatic effect against *Streptococcus aureus*[74]. But is has to be considered that depending on the type, bacteria demonstrate different high resistances against antibiotics (Table 4). Submucosal biofilm specimens were cultured from patients with peri-implantitis and after in vitro testing for susceptibility especially the combination of amoxicillin and metronidazole showed significant lower resistances (6.7%) [22].

**Table 4:** **Antibiotic resistance of *Prevotella intermedia*, *Prevotella nigrescens *** **and *Streptococcus constellatus *** **(n = 120) **[22]

**Antibiotic**	**Resistance**
Clindamycin	46,7%
Amoxicillin	39,2%
Doxycycline	25%
Metronidazole	21,7%
Amoxicilin & metronidazol	6,7%

Application of chlorhexidine resulted in the reduction of pocket depths, a higher implant adhesion and general weakening of inflammation measured by the level of the inflammatory markers IL-1 beta, VEGF and PGE-2 in various studies [75-77]. Compared to minocycline microsphere application repeated every three months [78], the treatment with 1% chlorhexidine gel resulted in significantly less reduced pocket depths after 12 months. Concerning tissue engineering, *Lan et al.* demonstrated a continuous release-kinetic of metronidazole for 30 days using a Poly-ϵ-Caprolacton/Alginat-ring [79]. *Hou et al.* incorporated fluorouracil into cylindrical poly-ϵ-caprolactone-implants of different diameters [80].

Local or systemic antibiotics are an additional therapy option. In combination with other conservative or surgical treatments it results in more efficient reductions of clinical peri-implantitis symptoms [81]. Just administration of antibiotics is no treatment option.

#### ***Laser therapy***

By means of a bactericide mode of action, CO_2_, Diode-, Er:YAG- (erbium-doped: yttrium-aluminum-garnet) and Er,Cr:YSGG- (erbium, chromium-doped: yttrium-scandium-gallium-garnet) lasers are used in the treatment of peri-implant diseases with increasing frequency. Minimal absorption and reverberations must be ensured with the purpose to protect implant and tissue. Er:YAG and Er,Cr :YAG with a wavelength of 3 microns can reduce biofilms up to 90% but in contrast to most mechanical therapies any biological compatibilities and cell stimulatory properties can’t be re-induced [5,82,83]. Treatment with a CO_2_ 308 nm excimer laser, however, led mainly and efficiently to satisfactory results in an anaerobic bacteria spectrum [84].

In comparison to mechanical methods (plastic curettes), treatments with an Er:YAG laser led to significantly better results in terms of bleeding at peri-implantitis. However, both methods showed no significant differences in changes of pocket depths, clinical attachment level, plaque index and gingival recessions, although in both groups these parameters were improved [85].

*Persson et al.* examined the effectiveness of Er:YAG lasers compared to an air polishing system in a randomized clinical trial with 42 patients over 6 months [86]. Except for different reducing effects on specific bacteria strains after one month (Er:YAG: *Fusobacterium nucleatum*; air polishing system: *Pseudomonas aeruginosa*, *Staphylococcus aureus* and *Peptostreptococcus anaerobius*) there were no long term-reducing effects shown after 6 months. In a recent study *Mailoa et al.* showed that laser therapy resulted in similar reductions of probing depths when compared to other decontamination methods [87]. Although there is only few data in comparison to manual and surgical therapy, laser therapy as a treatment option has to be considered as an adjunct. Further studies are needed to evaluate the profit of laser therapy in peri-implantitis treatment.

#### ***Photodynamic therapy***

Photodynamic therapy generates reactive oxygen species by multiplicity with help of a high-energy single-frequency light (e.g. diode lasers) in combination with photosensitizers (e.g. toluidine blue). In a wave length range of 580 to 1400 nm and toluidine blue-concentrations between 10 and 50 ug/ml, photodynamic therapy generates bactericide effects against aerobic and anaerobic bacteria (such as *Aggregatibacter actinomycetemcomitans, Porphyromonas gingivalis, Prevotella intermedia, Streptococcus mutans, Enterococcus faecalis*) [5,88,89]. The only prospective randomized clinical trail by *Basseti et al.* covered 12 months of follow-up. After manual debridement by titanium curettes and glycine air powder treatment half of the patients received adjunctive photodynamic therapy and the other half received minocycline microspheres into implant pockets. After 12 months, the number of periopathogenic bacteria and level of IL-1β decreased significantly in both groups without significant differences between them [73]. In a study by *Deppe et al.* regarding to the effectiveness of phototherapy on a moderate and severe peri-implantitis, both clinical attachment and bleeding index were significantly reduced suggesting that severe cases still resulted in bone resorption [90].

As a recommendation, photodynamic therapy has to be considered as an additional treatment option. Due to the fact that it is a relatively new approach, the data is rare and there are no long-term-studies available. Further evaluations and prospective clinical trials are needed for evaluation.

#### ***Surgical therapy***

The surgical therapy combines the concepts of the already mentioned non-surgical therapy with those of resective and/or regenerative procedures. The indication for the appropriate treatment strategy has been demonstrated in patient studies leading to the development of the “cumulative interceptive supportive therapy (CIST)” concept [91-93]. In 2004 it was modified and called AKUT-concept by *Lang et al.* (Table 5) [93]. The basis of this concept is a regular recall of the implanted patient and repeated assessment of plaque, bleeding, suppuration, pockets and radiological evidence of bone loss.

**Table 5:** **AKUT-protocol by *Lang et al. *** [93]

**Stage**	**Result**	**Therapy**
	Pocket depth (PD) < 3 mm, no plaque or bleeding	No therapy
A	PD < 3 mm, plaque and/or bleeding on probing	Mechanically cleaning, polishing, oral hygienic instructions
B	PD 4-5 mm, radiologically no bone loss	Mechanically cleaning, polishing, oral hygienic instructions plus local antiinfective therapy (e.g. CHX)
C	PD > 5 mm, radiologically bone loss < 2 mm	Mechanically cleaning, polishing, microbiological test, local and systemic antiinfective therapy
D	PD > 5 mm, radiologically bone loss > 2 mm	Resective or regenerative surgery

A further commonly accepted concept by *Zitzmann et al.* is referred to systematic periodontitis therapy. During the initial phase oral hygienic conditions have to be improved and mechanical cleaning and local antiinfective treatments are applied, if necessary. If non-surgical treatment fails, surgical intervention with open debridement and resective or regenerative therapy is recommended [3]. The concept of *Schmage* follows the CIST-protocol but recommends always mechanical and local disinfective treatments in stage A and B. Intervention should be performed if probing depths exceed 5 mm or are progressive as well as under occurrence of local inflammation signs [94].

#### ***Resective therapy***

**Figure 4:**
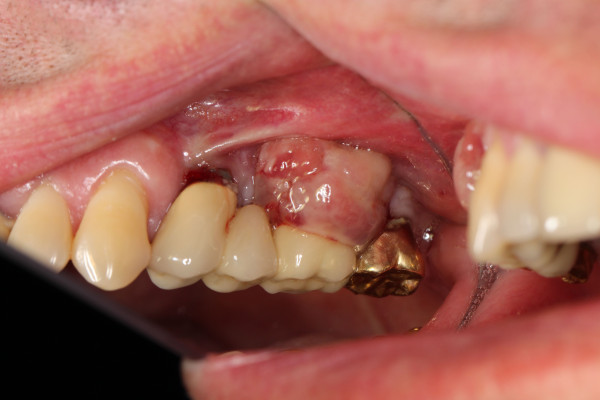
Peri-implantitis with granulation tissue.

**Figure 5:**
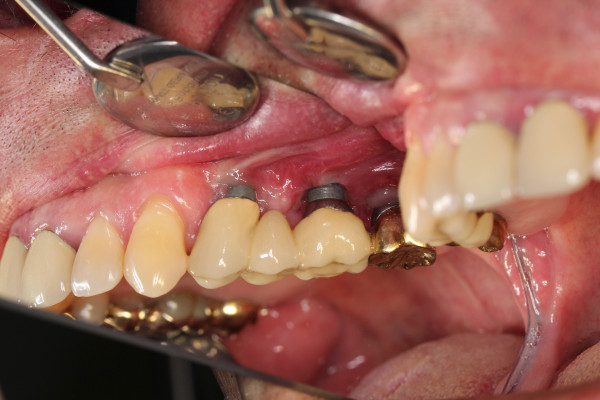
Peri-implantation 1 week after resective therapy.

In analogy to periodontitis, resective surgery has been shown to be effective in reduction of BOP, probing depths and clinical signs of inflammation. The basic principles include the elimination the periimplant osseous defect using ostectomy and osteoplasty as well as bacterial decontamination (Figures 4 and 5). Additionally, smoothening and polishing of the supracrestal implant surface (implantoplasty) may be applied.

*Serino et al.* showed that in patients with active peri-implant disease surgical pocket elimination and bone re-contouring in combination with plaque control before and after surgery represents an effective treatment. Two years after open reduction of inflammated peri-implant soft tissue and osseous surgery 48% of the patients had no signs of peri-implantitis and 77% of the patients had no implants with pocket depths ≥ 6 mm with bleeding and/or suppuration [56].

In a radiographic study with 3 years follow-up, *Romeo et al.* showed that the marginal bone loss after resective surgery with implantoplasty was significantly lower than after resective therapy only [55]. The group with additional implantoplasty also had significantly lower probing pocket depths, probing attachment levels and modified bleeding indices after 24 months [54].

Adjuvant implant surface decontamination with antimicrobial substances led to an initially less anaerobic bacteria contamination, but did not improve the clinical outcome [75].

Resective surgical therapy for peri-implantitis is a recommendable therapy option. Ostectomy and osteoplasty combined with implantoplasty represent an effective therapy to reduce or even stop peri-implantitis progression. Nevertheless, due to the increased postoperative recessions, this procedure is not suitable for every situation, especially in highly esthetic sensitive areas.

#### ***Regenerative approaches***

**Figure 6:**
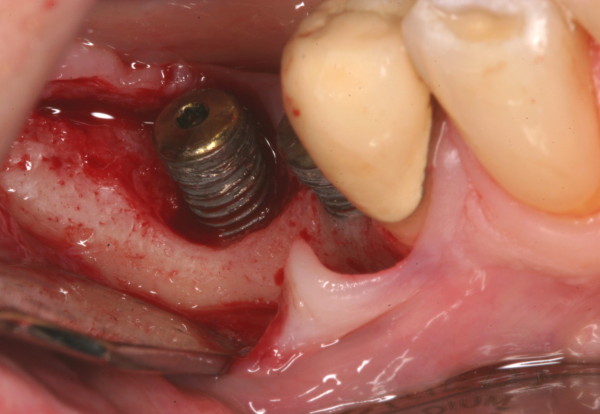
Regenerative therapie – defect after degranulation.

**Figure 7:**
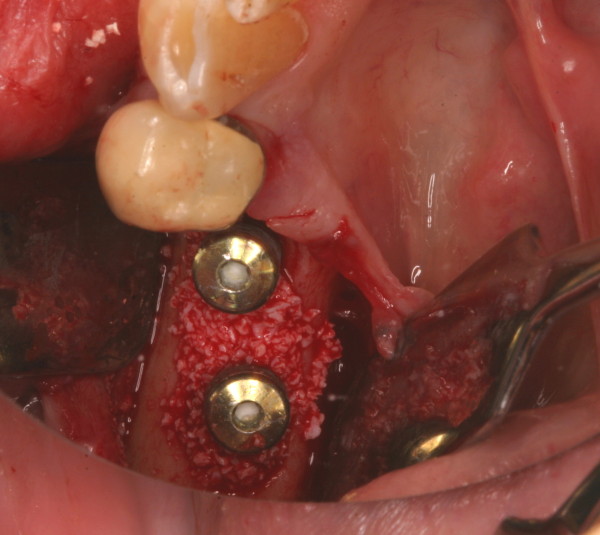
Regenerative therapie – defect fill with a xenograft material (BioOss ^®^, Geistlich, Switzerland).

**Figure 8:**
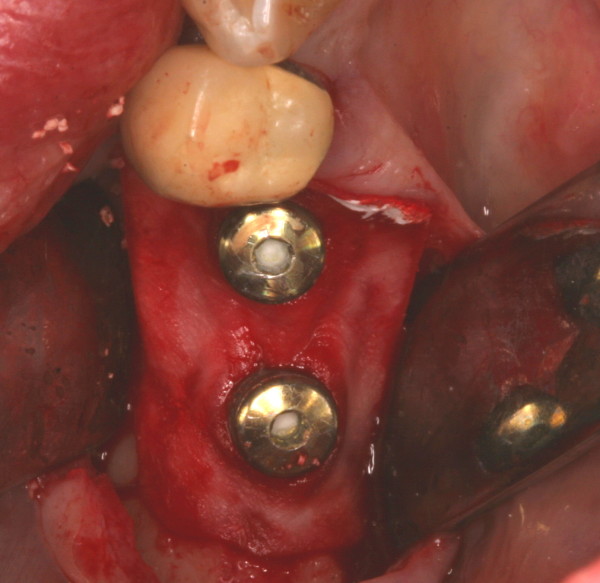
Regenerative therapie – membrane application (BioGide ^®^, Geistlich, Switzerland).

**Figure 9:**
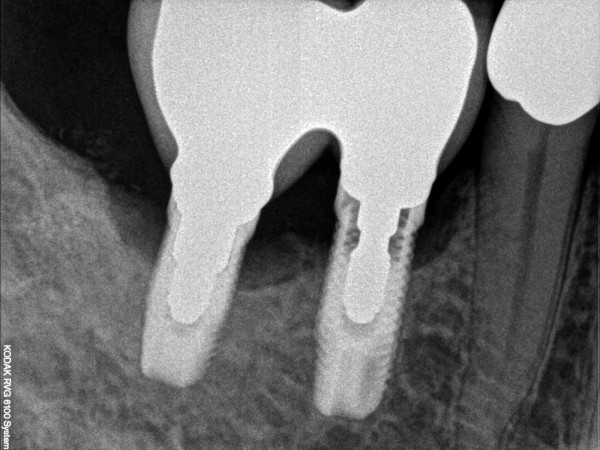
Preoperative radiograph of the peri-implant defect.

**Figure 10:**
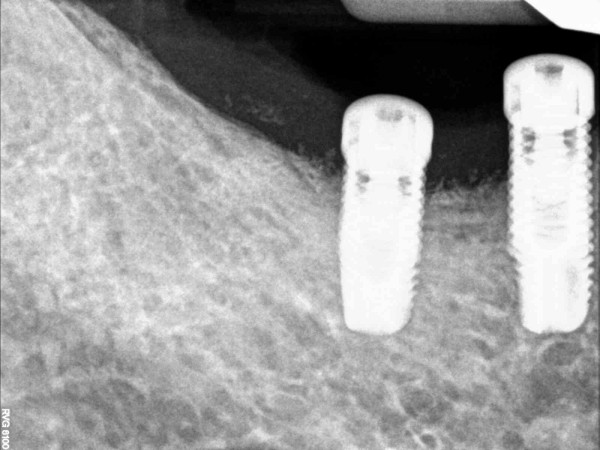
Postoperative radiograph 12 months after regenerative therapy.

Resective surgical therapy may result in re-osseointegration in only minor superficial defects. From functional, esthetic and long-time-survival point of views, full regeneration and re-osseointegration is aspired. In animal models it was possible to regenerate experimentally induced defects using various graft materials and/or resorbable membranes following the principles of guided bone regeneration (GBR) (Figures 6, 7, 8, 9 and 10).

In a study by *Hürzeler et al.* in 1997 in dogs, there was no significant difference between the application of membranes only versus membranes in combination with bone grafts (canine demineralized freeze-dried bone or hydroxyapatite) in terms of bone regeneration. However, the combination resulted in a greater amount of re-osseointegration [95]. No statistical differences in re-osseointegration could be demonstrated after treatment with GBR using a e-PTFE reinforced membrane compared to sites without this membrane [96]. The treatment resulted in 60–80% bone fill of the bony defect, but the absolute amount of re-osseointegration was small (between 0.1 - 0.6 mm).

In contrast to debridement with surface decontamination, in most of all animal studies regenerative methods were reported as more efficient. In general, GBR alone and bone fill alone have been shown to be more effective than debridement alone regarding to bone regeneration and re-osseointegration. The results of studies using a combination of membranes and bone graft materials were superior to those using membranes or bone grafts alone and tend to give the best results, However, there is a high variability in the amount of bone fill due to different investigation protocols and measurements [97-99] and not in all studies there was a benefit for these treatments compared to debridement alone [100-102]. The role of submerged healing in peri-implantitis has not been solved clearly. Although *Singh et al.* demonstrated in 1993 greater bone regeneration and re-osseointegration during submerged healing, *Grunder et al.* found no differences between either healing method [103,104].

Additionally, there are numerous studies regarding the treatment of peri-implantitis in humans under regenerative aspects. In a retrospective study of *Lagervall et al.* with 150 patients (382 implants) the most widely used operative intervention was the periodontal flap with osteoplasty (47%), followed by the use of bone replacement materials (20%). A cumulative success rate of 69% was recorded for both procedures, which was significantly lower in patients with risk factors such as smoking, periodontal disease and poor oral hygiene [29]. Regarding to a “regenerative” approach, autologous, allogenic and xenogenic bone replacement materials are often used for augmentation in bone defects used with or without collagen membrane. Allogenic and xenogenic grafts may be almost equivalent to autogenous material [105-107]. *Schwarz et al.* treated 22 patients randomly with access flap surgery and the application of nanocrystalline hydroxyapatite in contrast to xenogenic bone material with collagen membrane. No significant differences were determined between the groups, but 6 months after surgery both treatments resulted in clinically relevant reductions in probing depths and gains of clinical attachment level [108]. *Roos-Jansåker et al.* came to similar results using a coralline xenograft [19]. In another study bovine-derived xenogenic material was compared with autogenous bone as filling material for infracrestal defects. The xenograft provided radiologically more bone fill and decreases in pocket depths, while bleeding on probing and suppuration were observed at both procedures [109]. In a prospective study, 36 cases of peri-implant bone loss were treated after local disinfection and removal of granulation tissue with a 1:1 mixture of autologous bone and a xenogenic bone graft. The result was a mean radiologically reduction of 3.5 mm from 5.1 mm one year after treatment with an average reduction of probing depths of 4 mm [59]. In a recent prospective case series a combined resective and regenerative approach including a bovine bone mineral and a collagen membrane infracrestally and implantoplasty supracrestally showed a significant peri-implant probing depth reduction and an increased radiographic defect fill after 12 months of follow-up [110]. In another study of *Schwarz et al.* defect cleaning with either Er:YAG laser or plastic curettes/cotton pellets with saline was combined with regenerative surgical procedures (xenogenic bone substitute and collagen membrane). Thereby, the clinical outcome did not differ according to the chosen method of surface debridement [111].

In purpose of bone regeneration various approaches have been described with various success rates. There is a tendency that xenograft materials in combination with a resorbable membranes might have advantages in terms of re-osseointegration. Nevertheless, because of the lack of prospective randomized clinical studies there is no evident data concerning the long-time stability of such “defect fillings”.

## Conclusion

Due to the lack of prospective randomized long-term follow-up studies lots of approaches but no “ideal peri-implantitis therapy” have been described. There are many studies with different study designs in different populations with different materials used, but the sample sizes are often too small and the follow-up is too short. Therefore, prevention is the most important instrument based on appropriate treatment planning, an atraumatic approach for implant insertion and continuous check-up intervals with professional teeth and implant cleaning. Above all, attention should be paid to risk factors such as smoking and active or previous periodontitis. In non-surgical therapy, combinations of mechanical cleaning with curettes and air polishing systems are recommendable. Adjuvant antiseptic rinses and local or systemic antibiotics are effective for short-term bacteria eradication; laser and photodynamic therapy are additional treatment options. However, results for long-term benefits for these methods are missing.

Surgical therapy with resective and augmentative procedures completes the treatment options. Resective surgery can be used in order to eliminate peri-implant defects, to re-establish hygienic abilities and to reduce or even stop peri-implantitis progression. Regenerative approaches, e.g. with xenograft materials in combination with a resorbable membranes, are promising. The results of bone replacement materials and autologous bone grafts might be considered as nearly equivalent although long-term studies are still missing and only few studies with autologous bone material exist.

A graded systematic treatment planning according to the CIST protocol can be recommended. The “ideal peri-implantitis therapy”, actually, is a sum of approaches leading to an individual therapy regime concerning multifactorial etiology, treatment options and study results.

### Consent

Written informed consent was obtained from the patients for the publication of this report and any accompanying images.

## Competing interests

The authors declare that they have no competing interests.

## Authors’ contributions

The research has been carried out equally by RS, AH and OJ. JMS and MH supervised and corrected the manuscript. CH provided and edited the photographs. All authors read and approved the final manuscript.
